# Introversion and High Spatial Ability Is Associated With Origami Proficiency

**DOI:** 10.3389/fpsyg.2022.825462

**Published:** 2022-03-02

**Authors:** Mitsuhiko Hanada

**Affiliations:** Department of Complex and Intelligent Systems, Future University Hakodate, Hakodate, Japan

**Keywords:** origami, paper folding, spatial ability, personality, big-five model, introversion, extraversion

## Abstract

This study examined the relationship between origami performance, personality traits, and spatial ability. The researchers asked 43 Japanese university students (19 women and 24 men) to fold three models of origami (paper folding). Their performance was assessed by the number of successes in correctly folding the paper to make the models. They also answered the personality inventory NEO-FFI and completed the block-design test of the Wechsler Adult Intelligence Scale IV, which measures the spatial ability of people. The results showed that although origami performance demonstrated no significant relation with neuroticism, openness to experience, agreeableness, or conscientiousness, it improved as introversion tendency and spatial ability increased. There were no differences based on sex in origami performance. The findings suggest that performing origami requires spatial ability, which supports the view that origami is a potential educational material for training and enhancing spatial ability, and that introversion is advantageous to origami performance.

## Introduction

Origami is well known as the traditional Japanese practice of paper folding, although paper folding has been traditionally performed in other regions as well, such as China, Spain, and Germany (McArthur and Lang, 2013). Traditionally, there are a limited number of origami models; however, recently, a number of new origami models have been introduced. Origami is currently practiced worldwide. It is generally used for amusement, especially for children. There are many beautiful and complex origami figures that can be now considered a type of art (Lang, 2011; McArthur and Lang, 2013, 2017).

Origami has also been analyzed mathematically, and the computational aspects of origami, such as the computational complexity of flat folding, have been studied in the field of computational geometry (Demaine and O’Rourke, 2007). Origami is also used in engineering applications. For example, Miura-ori is famous for rigid origami folding, which has been proposed for the folding of solar panels in space (Hull, 2013).

Origami is also used in kindergarten schools and childcare in Japan to build a basic understanding of geometric shapes and concepts among children (Akimaru et al., 2007). Origami is popular in Japanese childhood education, probably because Fröbel, a German educator, who created the foundation of childhood education, included origami in his educational materials, and childhood education in Japan has been influenced by his education theory. He considered that origami can help children understand geometrical concepts intuitively (Igarashi, 2012). Furthermore, a number of attempts have been made to use origami for education in elementary and middle schools (Higginson and Colgan, 2001; Robichaux and Rodrigue, 2003; Boakes, 2009; Golan and Jackson, 2009; Wares, 2011, 2016; Arıcı and Aslan-Tutak, 2015; Burte et al., 2017), as well as in colleges (Boakes, 2011). Improvement of geometrical understanding by origami have been reported (Boakes, 2009, 2011; Arıcı and Aslan-Tutak, 2015; Burte et al., 2017). Origami has been considered to stimulate and train spatial ability while folding paper, that is, geometrically transforming a paper’s shape and making geometrical figures, which can contribute to the development of geometrical concepts (Boakes, 2009, 2011; Taylor and Hutton, 2013; Cakmak et al., 2014; Burte et al., 2020).

Although recent mathematical and technological analyses of origami have been actively explored, and origami has been used for education, psychological studies of origami have been rather scarce. However, several studies have examined the effects of origami on the improvement of spatial ability as well as geometrical understanding, and the developmental effect of spatial abilities were demonstrated for students in elementary schools (Taylor and Hutton, 2013; Burte et al., 2017), children with mathematical difficulty (Krisztián et al., 2015), middle- and high-school students (Boakes, 2009; Arıcı and Aslan-Tutak, 2015) and college students (Boakes, 2011). Moreover, training effects of origami on spatial ability was demonstrated on a study of neuroscience (Jaušovec and Jaušovec, 2012). Thus, origami has positive developmental and training effects on spatial ability. However, it is still unknown whether people with high spatial ability perform origami well. Physical exercise has good effects on cognitive abilities (Hötting and Röder, 2013), but good performers of physical exercise do not mean people with high cognitive abilities. Similarly, positive relationship between origami performance and spatial ability is not logically derived from positive effects of origami on spatial ability. Although aspects of spatial ability were measured by mental paper folding (Ekstrom et al., 1976), spatial ability may not be critical for origami because origami is a task of actually, but not mentally, folding paper. Thus, this study examined the relationship between origami performance and spatial ability. A positive relationship between origami performance and spatial ability would strengthen the claim that spatial ability is needed for origami and that origami is a potential educational material for training spatial ability.

Although origami is said to raise good emotional characteristics and personality such as carefulness and perseverance, very few studies have experimentally examined the psychological aspects related to origami. Hence, this study also examined the relationship between origami performance and personality. One of the famous factor models of personality is the Big Five model, which describes the personality of human beings using the five factors: neuroticism, extraversion, openness to experience, agreeableness, and conscientiousness (Digman, 1990; Goldberg, 1990). The Big Five model is supported for people from various cultural backgrounds, although variations across cultures have been noted (De Raad et al., 2010). The Big Five personality traits are related to creativity (Batey and Furnham, 2006; Furnham and Bachtiar, 2008; Furnham et al., 2008), and academic performance (O’Connor and Paunonen, 2007).

Origami is claimed to require cautiousness and perseverance because those engaged in it, called folders, have to carefully read origami diagrams and instructions and then follow instructions to fold models accurately. Most origami models cannot be folded if the order, location, and manner of folding are incorrect in the crucial folding steps. Cautiousness is generally regarded as a facet of conscientiousness, and perseverance is also related to this factor. If people need to be cautious and perseverant in performing origami, conscientious people would obtain a high score for origami performance. Conscientiousness is also associated with achievement-striving, and high conscientiousness is positively related to high academic success (Chamorro-Premuzic and Furnham, 2003; Noftle and Robins, 2007; O’Connor and Paunonen, 2007; Poropat, 2009). Further, conscientiousness is associated with higher performance in many domains of cognitive tasks in older adults (Sutin et al., 2019). This suggests that conscientiousness is positively related to origami performance.

Origami is also said to raise children’s creativity (Pope and Lam, 2009; Gur and Kobak-Demir, 2017); however, little empirical evidence has been presented to support this. Performing origami following instructions may appear to be unrelated to creativity, but it involves some aspects of problem-solving (Tenbrink and Taylor, 2015; Burte et al., 2017), and a high level of creativity would help in these aspects of problem-solving in origami. Studies have found that openness to experience has been related to creativity (Furnham et al., 2005) and cognitive flexibility (McCrae, 1987; Costa and McCrae, 2010). A study has found that openness is also associated with higher cognitive performance in older adulthood (Sutin et al., 2019). If creativity and cognitive flexibility help in performing origami, the performance of origami would be related to openness.

Extraversion/inversion is also related to cognitive performance; extraverts tend to be better at dividing attention to various tasks (Eysenck, 1982), resistance to distraction (Dornic and Ekehammar, 1990; Campbell, 1992; Dobbs et al., 2011), and retrieval from memory (Eysenck and Eysenck, 1979), whereas introverts perform better at vigilance (Shaw et al., 2010) and reflective problem-solving (Kumar and Kapila, 1987; Koelega, 1992). It is suggested that these associations of extraversion/introversion with cognitive performance are mediated through several psychological mechanisms, such as arousal and attentional control (Eysenck, 1982; Matthews, 2009). Although extraversion seems to be associated with better performance than introversion as a whole (Matthews, 2009), some studies reported that extraversion was negatively related to academic performance (Chamorro-Premuzic and Furnham, 2003; Furnham et al., 2003). Paper folding consists of many physical and mental operations, and extraversion/introversion is related to many cognitive functions, which may be related to extraversion/introversion in some ways, although it cannot be properly said which way it is.

Neuroticism has been reported to be related to poor cognitive performance (Eysenck, 1983; Eysenck and Eysenck, 1985) and was sometimes negatively associated with academic performance (Chamorro-Premuzic and Furnham, 2003; Poropat, 2009), mainly due to susceptibility to high anxiety and stress in people with high neuroticism (Eysenck, 1967; Eysenck and Eysenck, 1985; O’Connor and Paunonen, 2007). People with high neuroticism may feel stressed and anxious when they have to perform a task in the presence of an experimenter or examiner, and when their performance is going to be evaluated. Thus, those with high neuroticism might perform origami more poorly, although it would not be specific to origami. Agreeableness is not generally related to cognitive or academic performance (O’Connor and Paunonen, 2007). Hence, agreeableness can be said to be unrelated to origami performance.

The present study examined the relationship between origami performance and spatial ability and the Big Five personality traits. It formulated the following hypotheses that consider the above-discussed arguments: (1) As spatial ability increases, origami performance increases, (2) Conscientiousness and openness would be positively, and neuroticism would be negatively, related to origami performance; extraversion/introversion might be related to origami performance in some way, and agreeableness would not be related to origami performance. To test these hypotheses, we conducted an experiment in which participants answered questions on a Big Five personality inventory, took a test of spatial ability, and performed origami tasks. We also collected data on participants’ self-evaluations of school subjects and origami experiences to help interpret the data on the relationship between spatial ability, personality, and origami performance.

## Materials and Methods

### Participants

A total of 43 Japanese undergraduate and graduate students (20–24 years old, 19 women and 24 men) majoring in information sciences, participated in the experiment. The purpose of the experiment was withheld from the participants.

### Materials

#### Intelligence Test of Spatial Ability

The present study used the block-design test included in the Wechsler Adult Intelligence Scale (WAIS) IV (Wechsler, 2008) to measure spatial ability. In the task, participants arranged red and white blocks to match a displayed pattern. This test is considered to require spatial visualization ability and motor skills. It has been used for assessing spatial skills (Serbin and Connor, 1979; Casey et al., 2014).

#### Personality Inventory

This study used the Japanese version of the NEO-FFI Personality Inventory as a measure of participants’ personalities. The NEO-FFI is a short version of the revised NEO personality inventory (Costa and McCrae, 2010). It measures the Big Five personality traits of neuroticism, extraversion, openness to experience, agreeableness, and conscientiousness using 60 questions (12 for each trait).

#### Origami

**FIGURE 1:**
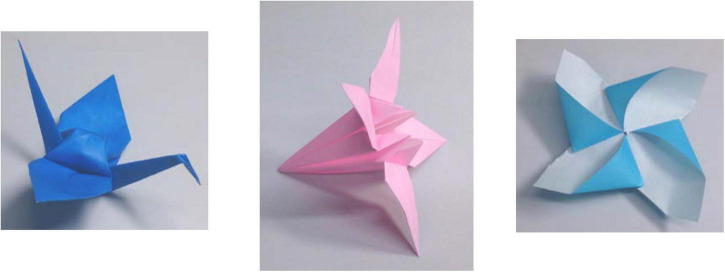
Photographs of origami figures for the origami tasks. The *crane* and *iris* are traditional Japanese origami models. The *windmill* was folded following the diagram and instructions in Haga’s book (Haga, 2014).

The participants were asked to fold the following three origami models: Crane, Iris, and Windmill. *Crane*: The crane is a traditional Japanese origami model known worldwide as a symbol of peace. *Iris*: The iris is also a traditional Japanese origami model, albeit not as popular as the crane, even among the Japanese. *Windmill*: This model was created by Haga (2014), who is famous for *origamics* (Haga, 2008), mathematical analysis of origami, and the application of origami to geometry education. The *windmill* is difficult to fold because it includes *tato* folding, a folding technique that requires the application of substantial tension on the paper [The *windmill* origami model of Haga (2014) is different from the well-known traditional windmill model and is much more difficult to fold.]. Images of the folded origami models are shown in Figure 1. The participants folded these models by looking at origami diagrams with Japanese instructions for each model. The diagrams and instructions for Haga’s *windmill* were copied from Haga’s book (Haga, 2014), whereas the diagrams for the *crane* and *iris* were taken from an Internet site (Takeuchi, n.d.).

#### Questionnaires

At the beginning of the experiment, the participants reported demographic information, such as age and sex, by answering a questionnaire. After completing the origami tasks, the participants filled out another questionnaire with the following questions (by selecting from the provided options): When were the first and last times you performed origami? (4–6 years old, 7–12 years old, 13–16 years old, 19–22 years old, never performed origami, or not remember when); How often do you fold origami? (3–4 days per week, 1–2 days per week, 1–2 times per month, 1–2 times per year, or never); What origami models are you good at folding? How well did you fold each of the origami models? (1: very poor to 7: very good), and How much did you enjoy folding each of the models (1: not at all to 7: very much). They were also asked about their proficiency in school subjects [Japanese, mathematics, foreign language (English), science, social studies, physical education, art, and music]. The participants responded by choosing the appropriate number (1: very poor to 7: very good), whether they like having fun outdoors (yes or no), and whether they like jigsaw puzzles (yes, no, neither yes nor no).

### Procedure

The experiment was conducted in a partitioned area of a quiet office room, and the participants completed the tasks and evaluations in the following order. (1) Answering questionnaire on demographic information; (2) Taking up WAIS block-design test; (3) Answering questions on Personality Inventory NEO-FFI; (4) Folding origami models in the following order: *crane*, *iris*, and *windmill*; and (5) Answering the final questionnaire. The participants folded the *crane* and *iris* models until they reported the completion of the folding or gave up on the completion of the models. A time limit of 20 min was set for folding the *windmill* model; otherwise, the experiment would have overextended if the participants were given as much time as they needed to decide to discontinue the process of folding the model. Origami performance was recorded using a video camera, although the data were not analyzed. The experiment lasted from 50 min to 2 h.

### Data Analysis

The participants were categorized into groups according to their performance level (i.e., the number of successes in the origami tasks). The study conducted MANOVA using Wilks Λ, with the scores in the personality trait scales and block-design test as dependent variables, and the origami performance group as an independent variable. Furthermore, to determine differences between the groups in detail, we conducted an ordered logistic regression analysis (Agresti, 2003), with the standardized scores in the personality trait scales and block-design test as the independent variables, and the performance group as the dependent variable, thus reversing the independent and dependent variables from those for the MANOVA. The data on self-evaluation of school subjects were analyzed similarly. The cross-tables of the origami performance and the results of the questionnaires were tested using Fisher’s exact test, with multiple comparisons corrected using Bonferroni’s method. All of the above analyses were conducted using R (R Core Team, 2020).

## Results

The cases in which the participants finished folding the origami models without any omitted steps in the instructions, regardless of the quality of the finished work, were categorized as successful folding, and those in which they gave up folding the paper to achieve the desired model or finished it incorrectly, were categorized as unsuccessful folding. Five types of success were identified: no success, success for only the *crane* model, success for only the *iris* model, success for the *crane* and *iris* models, and success for all three models. The number of participants for each of the five types is presented in Table 1. Eight participants (19%) correctly folded all the models. Twenty-four participants (56%) successfully folded the *crane* and *iris*, but not the *windmill*, and 11 (26%) successfully folded at most one model. Thus, we categorized the participants into three groups based on the number of successes in folding, namely, at most one, two, and three, to represent unskilled, ordinary, and proficient folders, respectively.

**TABLE 1:** Result patterns of the origami tasks.

Zero or one success			Two successes	Three successes
Zero	Only *crane*	Only *iris*	*Crane* and *iris*	*Crane*, *iris*, and *windmill*
2	3	6	24	8

*N = 43.*

**FIGURE 2:**
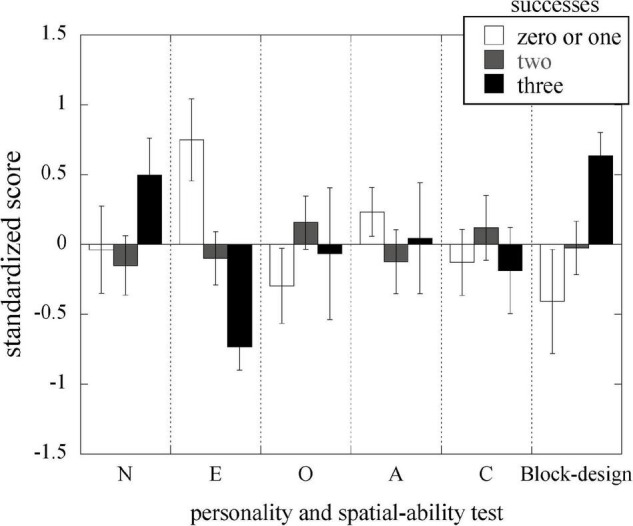
Mean standardized scores for personality traits in NEO-PPI and the block-design test of the WAIS-IV for each group, categorized based on the number of origami successes. The error bars represent standard errors. N, neuroticism; E, extraversion; O, openness to experience; A, agreeableness; C, conscientiousness.

The mean standardized scores of the five personality traits in NEO-FFI and the block-design test of WAIS IV for each of the three groups regarding origami performance are shown in Figure 2. The MANOVA on the standardized scores for the NEO-FFI and block-design test showed that the main effect of the groups was significant [Λ = 0.565, *F*(12,70) = 1.92, *p* = 0.046, η*_*p*_*^2^ = 0.248], indicating differences among the three groups. The results of the ordered logistic regression analysis are presented in Table 2. The order of the three groups was set according to the number of successes: “zero or one success,” “two successes,” and “three successes.” The coefficients for the extraversion and block-design test scores were found to be significant: the less extroverted (i.e., the more introverted) the participants were, the more they tended to succeed; the higher the score for the block-design test, the more they tended to succeed. These results were consistent with the mean scores of the groups (Figure 2). As the number of successes increased, the score for extraversion decreased, and the scores for the block-design test increased. The coefficients for neuroticism, openness to experience, agreeableness, and conscientiousness in the logistic regression analysis were not significant (*p* > 0.1).

**TABLE 2:** Ordinary logistic regression analysis of the origami successes with the scores for NEO-FFI and block-design test of the WAIS-IV.

	Coefficient	(Standard error)	*t*
Neuroticism	0.016	(0.040)	0.391
Extraversion	−0.204	(0.068)	−2.989**
Openness	0.055	(0.065)	0.841
Agreeableness	−0.002	(0.061)	−0.029
Conscientiousness	0.009	(0.056)	0.154
Block-design test	0.137	(0.061)	2.239*

Intercepts: g1| g2 = 2.07, g2| g3 = 5.70, *p < 0.05, **p < 0.01.

The sample size may be small for this logistic regression analysis. Maximum likelihood estimates of logit model coefficients have substantial bias in small samples. The number of events per variable (EPV), which indicates the number of outcome events per the number of candidate predictors, are generally recommended to be 10 or greater (Peduzzi et al., 1996). However, this rule would not be applied in that analysis. The expiatory variables for that regression analysis are not correlated so much. The correlation coefficients between expiatory variables were less than 0.3 except the correlation between conscientiousness and openness (0.359). It is unsurprising, because the Big-Five personality traits are known to be fairly independent of each other, and spatial ability. In the case that explanatory variables are uncorrelated, we can approximately estimate the effect of each variable separately, with one explanatory variable. For just one explanatory variable, a much smaller number of samples is required. Thus, the required sample size would be much smaller than that suggested by the EPV of 10 for that analysis. Still, the number of the samples are fairly small. Since it was reported that Firth (1993) penalized maximum likelihood estimator [equivalently, maximum penalized likelihood with powers of the Jeffreys (1946) prior as penalty] instead of maximum likelihood estimator eliminates most of the bias in the logistic regression due to small samples (Rainey and McCaskey, 2021). Hence, the ordered logistic regression was conducted by the method of Firth’s maximum penalized likelihood using R package *brglm2* (Kosmidis et al., 2020; Kosmidis, 2021). The results were similar to those by the maximum likelihood method, though the regression coefficients were a little smaller than the ones shown in Table 2 as a whole. The regression coefficient of extraversion was still significant (*p* = 0.012), and that of spatial ability was almost significant (*p* = 0.052), while the other regression coefficients were not significant (*p* > 0.10). Thus, the results of this logistic regression were fairly robust.

The scores for the self-evaluation of the school subjects were similarly analyzed. The mean standardized scores for each of the three groups regarding origami performance are shown in Supplementary Figure 1. The MANOVA of the standardized scores for school subjects showed a significant main effect of the group [Λ = 0.443, *F*(16,66) = 2.07, *p* = 0.020, η*_*p*_*^2^ = 0.32]. The results of the ordered logistic regression with the standardized scores as independent variables and the grouping as the dependent variable are shown in Supplementary Table 1. Only the coefficient for physical education was found to be significant: as the self-evaluation scores for physical education decreased, the origami performance improved. The ordered logistic regression was also conducted by the method of Firth’s maximum penalized likelihood to this data. Although the regression coefficients became smaller on the whole, the results were similar to that of the maximum-likelihood logistic regression. Only the coefficient of physical education was significant (*p* = 0.031).

Table 3 shows the cross-tabulation of performance (zero or one, two, and three successes) × sex of the participants (female and male), frequency of performing origami, and the last time the participants performed origami. As some cells in the tables had very small numbers, some response categories regarding the frequency of performing origami and the last time of origami folding were integrated. Fisher’s exact test correcting multiple comparisons using Bonferroni’s method, which tested the independence of origami performance, sex, frequency, and the last time of origami folding, showed that sex and frequency of performing origami were not significantly related to origami performance (*p* > 0.1). Meanwhile, the last time of origami folding was related to origami performance (*p* < 0.05); skilled origami performers tended to have performed origami more recently.

**TABLE 3:** Number of origami successfully folded crossed by sex, frequency of performing origami, and last time of origami folding.

	Number of origami successfully folded		
	Zero or one	Two	Three
**Sex**			
Female	4	11	4
Male	7	13	4
**Frequency of origami folding**			
Sometimes	3	11	6
Never	8	13	2
**Last time of origami folding**			
Earlier than university entrance	8	10	1
Later than university entrance	2	11	7
Not remember	1	3	0

## Discussion

This study examined the relationship between origami performance, personality traits, and spatial ability. The participants folded three models of origami, and their performance was assessed by the number of successes in folding the models correctly. They also took the personality inventory NEO-FFI and completed the block-design test of the WAIS IV, which measures spatial ability. The results showed that origami performance improved as introversion tendency and spatial ability increased; however, it demonstrated no significant relationship with neuroticism, openness to experience, agreeableness, or conscientiousness. The findings suggest that performing origami requires spatial ability and that introversion is advantageous to origami performance.

The study did not score the quality of the folded origami owing to the difficulty of achieving an objective scoring. We tried to make the standard for evaluating the quality of folding for each model, and scoring the folded figures. However, it was found to be difficult to rate the quality of the folded figures objectively and there remained some subjective elements in the evaluation. Moreover, the quality of almost all the correctly folded origami figures was fairly high, and was not varied so much. On the other hand, whether origami folding is correctly accomplished can be determined objectively to a fair degree. Therefore, this measure of performance evaluation for origami folding would be appropriate. However, relationship between the quality of folded origami figures and spatial ability/personality would be an interesting issue. How to evaluate the folded figures objectively is an issue for the future.

Spatial ability was positively related to origami performance; good/poor origami performers have higher/lower spatial abilities. The need and use of spatial ability while folding paper physically, but not mentally following origami instructions, is yet unknown and there is a lack of clear empirical evidence. The positive relationship between origami performance and spatial ability shown in this study strongly suggests that spatial ability is needed to perform origami correctly. Prior studies have found that origami has been used to improve spatial ability and geometrical understandings (Higginson and Colgan, 2001; Robichaux and Rodrigue, 2003; Boakes, 2009, 2011; Golan and Jackson, 2009; Wares, 2011, 2016; Burte et al., 2017). The findings of this study provide the basis for the use of origami to improve spatial ability, although further studies should be conducted on how to use origami as educational materials, and actual training effects of origami on the improvement of spatial ability.

The study hypothesized that extraversion/introversion might be associated with origami proficiency in some way. The results indicated that the more introverted the participants were, the better they were at folding the origami models. Since the reported relations of extraversion/introversion to performance of cognitive tasks depend on the task itself, extraverts are better at dividing attention to various activities (Eysenck, 1982), resisting distraction (Dornic and Ekehammar, 1990; Campbell, 1992; Dobbs et al., 2011), and retrieving from memory (Eysenck and Eysenck, 1979), whereas introverts perform better in vigilance (Shaw et al., 2010) and reflective problem-solving tasks (Kumar and Kapila, 1987; Koelega, 1992). The origami tasks of this study should not demand divided attention and retrieval from memory so much, because the participants folded paper reading the instructions. However, some vigilance would be required for folding paper, and some amount of reflective problem solving is needed to translate the origami instructions to actual physical folding. Thus, introverts may benefit from high vigilance and reflective problem-solving abilities. Alternatively, the difference in origami performance between introverts and extraverts may be explained by the different ways in which they engage in social interactions. Extraverts like communicating with other people, whereas introverts tend to prefer activities without social interaction (Kirkcaldy and Furnham, 1991). Hence, introverts may be more familiar with activities performed alone, such as reading books compared with extraverts (Eysenck and Eysenck, 1964). Origami is an activity that is usually performed alone, which may be ideal for introverts. In addition, introverts may have more experience in origami folding, probably because it can be performed alone, whereas extroverts may have fewer experiences, probably because they prefer social activities such as team sports to origami (Eagleton et al., 2007). This explanation is consistent with the results shown in Table 3 that skillful folders tended to have performed origami more recently. Introverted people may be relatively poor at social interactions, but good at non-social activities, and thus, their introversion would prove to be advantageous for performing origami. It is noteworthy that this relation between origami performance and extraversion cannot be explained by the correlation between spatial ability and extraversion as the correlation between the scores of the block-design test and extraversion trait was extremely weak (*r* = −0.073), and the effect of extraversion on origami performance shown in the ordered logistic regression (Table 2) did not include those of the other explanatory variables, including extraversion. Furthermore, no significant relationship between spatial intelligence and extraversion was reported (Roberts, 2002).

The study also hypothesized that openness and conscientiousness are related to origami performance. The results showed no correlations, which is rather surprising, considering the clearly observed relationship between introversion and origami performance. The lack of relationship between conscientiousness and origami performance may be explained by the long-term nature of personality; carefulness and perseverance, which are components of conscientiousness, and was hypothesized to contribute to origami performance, would be rather long-term tendencies of behaviors (Duckworth et al., 2007), and it might not reflect the short-term mental concentration needed to perform our origami task. Alternatively, lack of care and perseverance may not be a primary cause of origami failure; those traits would not contribute to high origami performance if a participant did not understand how to fold the origami models in the first place.

Although we hypothesized a positive relationship because openness is related to creativity, it was found that openness was not related to origami performance. Openness was reported to be related to divergent thinking (McCrae, 1987), which is considered the basis of creativity. Performing origami following instructions should be a type of problem- solving in which well-defined solutions exist. Hence, convergent, but not divergent thinking, is important for origami. Since openness is not a good indicator of convergent thinking ability, it might not reflect origami performance efficiently. The lack of a relationship between openness and origami performance may also be explained by the view that creativity is not a single construct and may consist of several aspects of creativity, which are related to different cognitive abilities and styles. A study reported that artistic, scientific, and verbal creativity can be separated, and object visualization is related to artistic creativity and spatial visualization is associated with scientific creativity (Kozhevnikov et al., 2013). Artists seem to have higher openness than scientists, although highly creative scientists tend to have higher openness than non-creative ones (Feist, 1998). Hence, openness is related more to artistic creativity than to scientific creativity. Since origami performance following instructions is associated with spatial ability, it may relate to scientific creativity, but it may not be related to verbal and artistic creativity (However, artistic creativity should be needed for the creation of new origami models.). The weak relationship between openness and scientific creativity may explain the lack of a significant relationship between origami performance and openness.

We also hypothesized that neuroticism is negatively related to origami performance due to its susceptibility to mental stress. However, in this study, it was noted that neuroticism was higher for good origami performance than for poor performance (Figure 2), although this difference in origami performance was not statistically significant. Since our origami task is like a play activity, but not like a cognitive or academic test, the task may not invoke mental stress even to people with high neuroticism. Alternatively, origami may not be affected by stress levels. Measurement of the stress level during or after origami would clarify which of these explanations is correct.

Origami is becoming increasingly popular worldwide, with many exhibitions of artistic origami works in recent times (McArthur and Lang, 2013, 2017). Moreover, the mathematical and computational aspects of origami have been analyzed in the fields of mathematics and technology by various studies (Demaine and O’Rourke, 2007; Hull, 2013). Meanwhile, studies on origami in the field of psychology have been scarce. The present study showed that introversion and spatial ability are related to origami performance. In the area of education, origami is popular in Japan’s kindergarten and childcare facilities, and it is attracting attention in other countries. Many attempts have been made to improve spatial ability and geometric understanding using origami as a mathematical material (Boakes, 2009, 2011; Taylor and Hutton, 2013; Cakmak et al., 2014). Introverted people are observed to be good at origami; they may be more interested in the geometry of paper folding than extraverts. Mathematical education by origami may be more appropriate for introverted people than for extraverts. Whether the efficacy of geometrical education using origami differs between extraverts and introverts would be an issue to be addressed through future studies.

This study had several limitations. First, all of the participants were young university students; the findings on the relationship between origami performance, extraversion, and spatial ability may not apply to children and older populations. Second, the participants were Japanese, and almost all Japanese had some experience in origami folding during their childhood. Thus, the present results may not be generalizable to people from different cultural backgrounds and with different education levels. Third, the study used three origami models for origami tasks, but there may be more appropriate origami models for studying the relation between origami performance, spatial ability, and personality. Although further studies are required to examine these issues, the present work provides the first step in elucidating the psychological aspects of origami.

## Data Availability Statement

The raw data supporting the conclusions of this article will be made available by the authors, without undue reservation.

## Ethics Statement

The studies involving human participants were reviewed and approved by the Ethics Committee of Future University Hakodate. The patients/participants provided their written informed consent to participate in this study.

## Author Contributions

The author confirms being the sole contributor of this work and has approved it for publication.

## Conflict of Interest

The author declares that the research was conducted in the absence of any commercial or financial relationships that could be construed as a potential conflict of interest.

## Publisher’s Note

All claims expressed in this article are solely those of the authors and do not necessarily represent those of their affiliated organizations, or those of the publisher, the editors and the reviewers. Any product that may be evaluated in this article, or claim that may be made by its manufacturer, is not guaranteed or endorsed by the publisher.
