# Supplementary material for: Introversion and High Spatial Ability Is Associated With Origami Proficiency
Source: Front Psychol. 2022 Mar 2;13:825462. doi: 10.3389/fpsyg.2022.825462 (PMC8924060; doi:10.3389/fpsyg.2022.825462)
Supplement: Supplementary file 1 [file Data_Sheet_1.pdf]

Supplementary material

Table S1. Ordinary logistic regression of the origami successes to the subjective evaluation scores of school subjects

	Coefficient	(Standard error)	t	
Japanese language	-0.428	(-0.368)	-1.165	
Mathematics	-0.308	(-0.380)	-0.811	
Foreign language (English)	-0.140	(-0.275)	-0.508	
Science	0.640	(-0.361)	1.771	
Social studies	-0.278	(-0.262)	-1.058	
Physical education	-0.726	(-0.290)	-2.502	*
Art	0.380	(-0.304)	1.250	
Music	0.288	(-0.339)	0.849	

Intercepts: g1|g2 = -3.92, g2|g3 = -0.396, * $p < 0.05$, ** $p < 0.01$

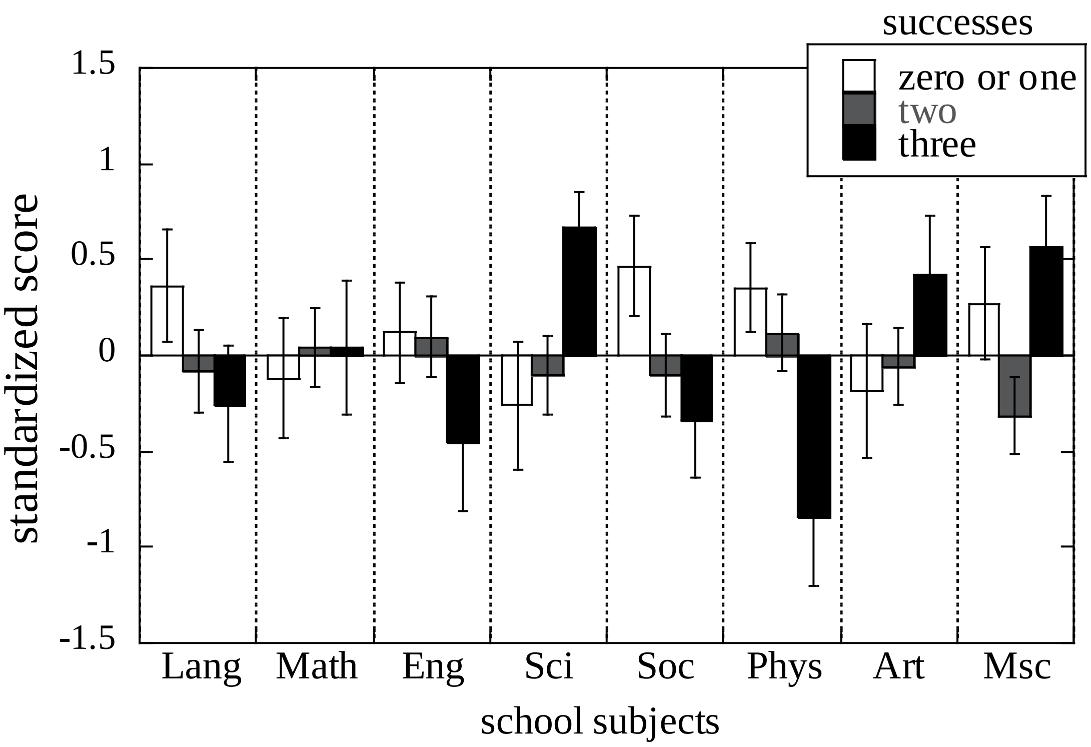

Figure S1. Mean standardized scores for proficiency in school subjects evaluated by the participants themselves, categorized based on the number of origami successes. The error bars represent standard errors. Lang: Japanese language, Math: mathematics, Eng: foreign language (English), Sci: science, Soc: social studies, Phys: physical education, Art: art, Msc: music.
